# Prognostic and Risk Stratification Value of Lesion MACC1 Expression in Colorectal Cancer Patients

**DOI:** 10.3389/fonc.2019.00028

**Published:** 2019-02-05

**Authors:** Aifen Lin, Xia Zhang, Rui- Li Zhang, Xiao-Fang He, Jian-Gang Zhang, Wei-Hua Yan

**Affiliations:** ^1^Biological Resource Center, Taizhou Hospital of Zhejiang Province, Wenzhou Medical University, Linhai, China; ^2^Department of Gastrointestinal Surgery, Taizhou Hospital of Zhejiang Province, Wenzhou Medical University, Linhai, China; ^3^Department of Laboratory Medicine, Lanxi Peoples's Hospital, Lanxi, China; ^4^Medical Research Center, Taizhou Hospital of Zhejiang Province, Wenzhou Medical University, Linhai, China

**Keywords:** MACC1, colorectal cancer, survival, prognosis, risk stratification

## Abstract

The up-regulated metastasis-associated in colon cancer 1 (MACC1) expression and its clinical significance has been explored in a varity of malignancies. In this study, lesion MACC1 expression in 503 CRC patients (*N*_colon_ = 332, *N*_rectal_ = 171) were analyzed with immunohistochemistry, and its correlation with clinical parameters, patient survival, and its impact on prognostic stratification were evaluated. Data revealed the survival of patient with MACC1_high_ is markedly worse than that of MACC1_low_ (mean overall survival: 80.1 vs. 90.4 months; *p* = 0.001) and is an independent prognostic predictor (hazard ratio = 1.533; *p* = 0.005). More importantly, for the first time, we demonstrated that MACC1 status exhibited a significantly prognostic power for stratified clinical parameters such as patient age and gender, particular TNM status, and distinct AJCC disease stage. In summary, our findings indicated that MACC1 is a valuable prognostic and risk stratification biomarker for colorectal cancer patients.

## Introduction

Cancer is one of the major public health problems and the leading cause of death worldwide. In this scenario, the incidence and mortality of the colorectal cancer (CRC) has been observed on an upward trend during the last decade in China. There were 376,300 new CRC cases and 191,000 deaths estimated for the year of 2015, making CRC among the most commonly diagnosed and cancer-related death in our country (1, 2).

Cancers have developed comprehensive strategies such as aberrant induction of cancer-promoting molecule expression to counteract host anti-tumor responses for malignant cell survival and metastasis, and finally result in disease progression (3). Among tremendous kinds of tumor promoting antigens, metastasis-associated in colon cancer 1 (MACC1) has been found to play pivotal roles in cancer tumorigenesis and metastasis, and its relevance in chemoresistance has also been reported (4–7).

In CRC patients, it has been found that tumor lesion MACC1 expression significantly increased in relation to its non-tumorous adjacent tissues. Enhanced MACC1 expression has been reported to be significantly related to tumor metastasis and worse disease outcome, and to be an early risk factor for cancer patients (8, 9). Moreover, higher circulating MACC1 transcripts and soluble MACC1 proteins have also been found relating to unfavorable prognosis for cancer patients (10, 11). Other than CRC, as it has been investigated, the prognostic value of MACC1 has been further proved in other malignancies such as hepatocellular cancer (12), ovarian cancer and breast cancer (13, 14). In this context, lesion MACC1 expression has been suggested as a poor prognostic biomarker in patients with non-small cell lung cancer (15). Tan et al. (11) reported that serum MACC1 levels can not only discriminate breast cancer patients from normal controls, but can also have high MACC1 levels that is related to patient worse disease-free survival. Burock et al. (10) presented that peripheral circulating MACC1 transcripts is a valuable diagnostic and prognostic factor for gastric cancer patients.

However, being the heterogeneity of cancers, the disease outcome can vary markedly even among patients with the same tumor-lymph nodes-metastasis (TNM) status or The American Joint Committee on Cancer (AJCC) stages (16). Therefore, in order to improve disease outcome prediction in distinct subpopulations of cancer patients, the importance of risk stratification with other prognostic factors has to be recognized (17, 18). In this study, we assessed the impact of lesion MACC1 expression on survival and prognostic stratification value in a large cohort of 503 CRC patients.

## Patients and Methods

### Colorectal Cancer Patients

503 CRC patients were consecutively included between April 2007 and May 2013, with the median age of 66 years (range from 19 years to 90 years). All patients were diagnosed at the Department of Gastrointestinal Surgery, Taizhou Hospital of Zhejiang Province, China. The samples were provided by the Tissue Bank of Taizhou Hospital of Zhejiang Province (National Human Genetic Resources Platform of China YCZYPT [2017]02). A written form of consent was obtained from each participant prior to the surgery, and this study was approved by the Institutional Ethics Review Board of Taizhou Hospital of Zhejiang Province.

Clinical disease stage was determined with the AJCC 7th TNM staging system (19). Follow-up data were available for 499 patients until the last follow-up on December 2016. The median follow-up was 53.0 months (range: 3~137 months), and 180 deaths of the CRC patients occurred during the period. Patient overall survival was calculated from the date of surgical operation to the last follow-up. Clinicopathological details of the CRC patients are listed in Table 1.

**Table 1:** Association between MACC1 status with clinicopathological parameters in colorectal cancer patients.

**Variables**	**No. cases**	MACC1 (median)^**^		p^*^	MACC1 (cut-off)^**^		p^*^
		**Low (%)**	**High (%)**		**Low (%)**	**High (%)**	
CRC patients	503	282 (56.1)	221 (43.9)		405 (80.5)	98 (19.5)	
**TYPE**							
Colon	332	175 (52.7)	157 (47.3)	0.037	257 (77.4)	75 (22.6)	0.017
Rectal	171	107 (62.6)	64 (37.4)		148 (86.6)	23 (13.4)	
**GENDER**							
Male	290	173 (59.7)	117 (40.3)	0.069	237 (81.7)	53 (18.3)	0.428
Female	213	109 (51.1)	104 (48.9)		168 (78.9)	45 (21.1)	
**AGE**							
≤ 66 years	261	154 (59.0)	107 (41.0)	0.178	214 (82.0)	47 (18.0)	0.431
>66 years	242	128 (52.9)	114 (47.1)		191 (79.0)	51 (21.0)	
**T CATEGORY**							
T_1+2_	223	132 (59.2)	91 (40.8)	0.427	191 (85.6)	32 (14.4)	0.001
T_3_	260	140 (53.8)	120 (46.2)		203 (78.1)	57 (21.9)	
T_4_	20	10 (50.0)	10 (50.0)		11 (55.0)	9 (45.0)	
**N CATEGORY**							
N_0_	274	162 (59.1)	112 (40.9)	0.468	226 (82.5)	48 (17.5)	0.468
N_1_	144	77 (53.5)	67 (46.5)		112 (77.8)	32 (22.2)	
N_2_	85	43 (50.6)	42 (49.4)		67 (78.8)	18 (21.2)	
**M CATEGORY**							
M_0_	487	276 (56.7)	221 (45.3)	0.202	395 (81.1)	92 (18.9)	0.064
M_1_	16	6 (37.5)	10 (62.5)		10 (62.5)	6 (37.5)	
**AJCC STAGE**							
I	147	89 (60.5)	58 (39.5)	0.201	127 (86.4)	20 (13.6)	0.066
II	121	71 (58.7)	50 (41.3)		96 (79.3)	25 (20.7)	
III	219	116 (53.0)	103 (47.0)		172 (78.5)	47 (21.5)	
IV	16	6 (37.5)	10 (62.5)		10 (62.5)	6 (37.5)	
**PATIENT STATUS**							
Alive	319	195 (61.1)	124 (38.9)	0.004	277 (86.8)	42 (13.2)	< 0.001
Died	180	85 (47.2)	95 (52.8)		124 (68.9)	56 (31.1)	

* *Comparison of MACC1 expression status between or among each variable using the Pearson chi-square test or Fisher's Exact test. TNM, lymph-node-metastasis and stage*.

** *MACC1 index (median) = 0.950 and (cut-off) = 1.04*.

### Immunohistochemistry (IHC) and Evaluation of Staining

Immunohistochemistry were performed with 4 μm sections of paraffin-embedded tissues on polylysine coated slides. Slides were deparaffinized with xylene and rehydrated through gradient ethanol. Antigen retrieval was performed with 10 mM sodium citrate buffer (pH 6.0), and endogenous peroxidase activity was blocked with 3% H_2_O_2_. Then incubated with anti-MACC1 mAb (CL0856) (1:500, Thermo Fisher, Rockford, IL, USA) overnight at 4°C. After they were thoroughly washed with 0.01 M phosphate-buffered saline (PBS), incubated with Envision anti-mouse and visualized by DAB development with a Dako EnVison kit (Dako, Glostrup, Denmark). Finally, all slides were counterstained with hematoxylin and mounted with glycerol gelatin.

MACC1 expression index was assessed according to the intensity of staining and percentage of the MACC1 positive tumor cells. The staining intensity and proportion of MACC1 expression were scored independently by two observers who did not have access to the patients' information, and an average score of all samples were obtained. The staining intensity score was defined as: 0 (no staining); 1 (weak to moderate staining), and 2 (strong staining). MACC1 index of each slide was determined by multiplying the score of staining intensity and the percentage of MACC1 expression. The tumor MACC1 expression index in this study ranges 0~2.

**Figure 1:**
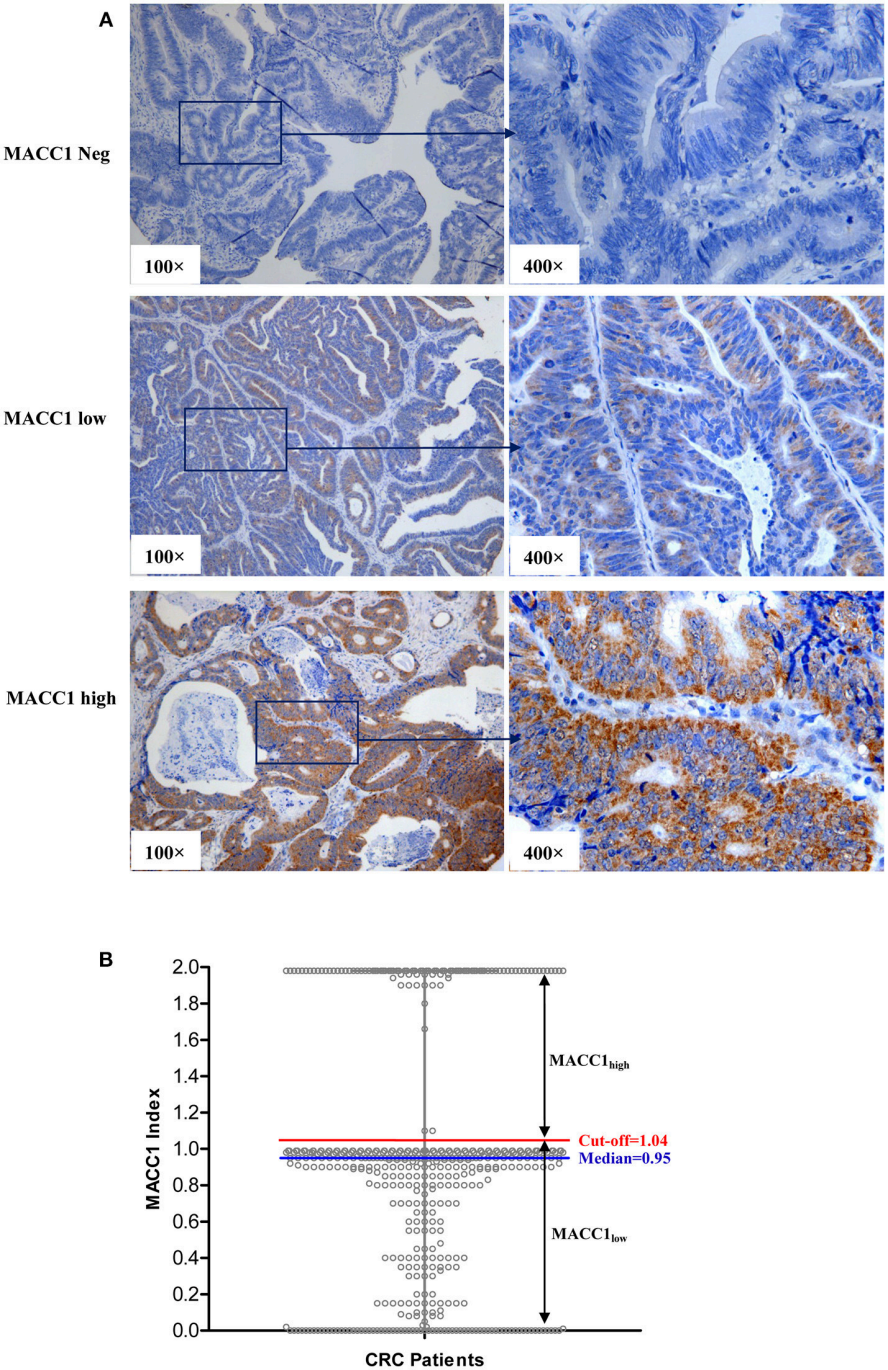
**(A)** Representative immunohistochemistry staining of MACC1_neg_ (index = 0), MACC1_low_ (index = 0.75), and MACC1_high_ (index = 1.96) expression in primary CRC sections. **(B)** The distribution of the index of MACC1 expression in CRC patients. The red line represents the cut-off value determined by ROC curve. The blue line represents the median of the index of MACC1 expression.

The representatives of immunohistochemistry are shown in Figure 1A and the distribution of the index of MACC1 expression is shown in Figure 1B. The range of the index was from 0 to 1.98 (median = 0.950). In addition to the median level, an appropriate cut-point determined by the receiver operating characteristic (ROC) curve to stratify the MACC1_high_ and MACC1_low_ groups was performed as recommended by Rohr et al. (20). In this study, the optimum cut-off for strata of MACC1 expression was determined by the ROC curve (between the CRC patients who survived and died) with the maximum of sensitivity and specificity according to the Youden's Index, and an optimum cut-off = 1.04 was obtained for the cohort (Figure 1B).

Therefore, in this study, two thresholds of MACC1 expression (median and cut-off) were analyzed for the CRC patient survival. Patients with MACC1 index> (median = 0.950) were defined as MACC1_high_ and ≤ 0.950 as MACC1_low_. Moreover, we further analyzed the patients whose MACC1index >ROC based (cut-off = 1.04) were defined as MACC1_high_ and ≤ 1.04 as MACC1_low_.

### Statistical Analysis

Statistical analysis was performed with the SPSS 13.0 statistical software package (SPSS, Inc., Chicago, IL, USA). ROC curve was performed and the cut-off value was determined by Youden's index. The relationship between MACC1 status and CRC patient clinicopathological parameters were performed with Fisher's exact test or Chi-square test. Survival curves were performed with the Kaplan-Meier method, and differences between survival were compared with the log-rank test. The significance of variables for survival was conducted with the Cox proportional hazards model in multivariate analysis. *P* < 0.05 (two-tailed) was considered statistically significant.

## Results

### Relationship Between MACC1 Levels and Clinical Variables in CRC Patients

According to the median level of MACC1 index (median = 0.950), among 503 CRC patients, there were 221 MACC1_high_ and 282 MACC1_low_ CRC patients, respectively. Based on the optimum cut-off by the Youden's Index, there were 98 MACC1_high_ and 405 MACC1_low_ CRC patients in this study.

The relationship between MACC1 expression and clinical variables of CRC patients is detailed in Table 1. The data revealed that patients with MACC1_high_ were more frequently observed in colon cancer patients than those in rectal cancer patients with both thresholds (*p*_median_ = 0.037 and *p*_cut−off_ = 0.017). The higher percentages of MACC1high were also observed in the CRC patients that had died (*p*_median_ = 0.004 and *p*_cut-*off*_ < 0.001). Among the whole cohort of CRC patients, an increasing trend of MACC1_high_ were found among CRC patients with advanced pT, pN, pM categories and advanced AJCC disease stages (Table 1).

### MACC1 Levels Related to Survival in CRC Patients

**Figure 2:**
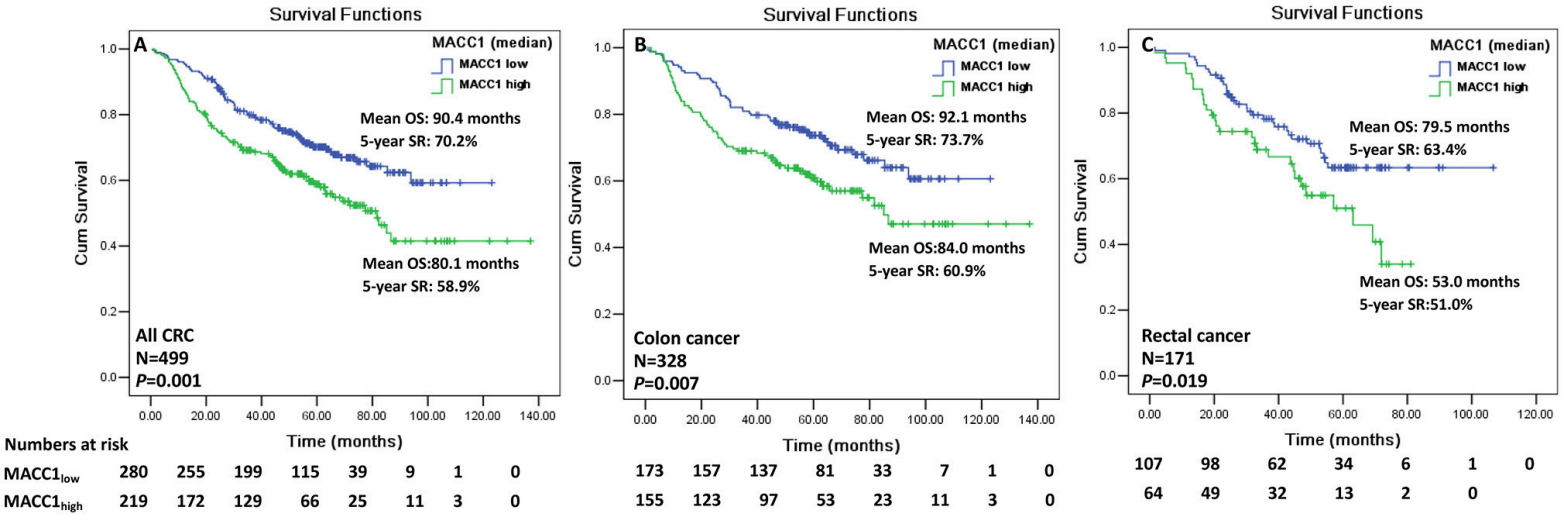
Kaplan-Meier survival analysis of the index of MACC1 expression with the median (0.950) for CRC patients. Comparison of overall survival between MACC1_low_ and MACC1_high_ among **(A)** all CRC patients; **(B)** colon cancer patients; and **(C)** rectal cancer patients.

We, then evaluated the clinical significance of MACC1 levels to survival in CRC patients. Data showed that CRC patients with MACC1_high_ (>median) have a significantly worse overall mean of survival (OS) than those with MACC1_low_ (80.1 vs. 90.4 months), and the 5-year survival rate (SR) for the two groups is 58.9 vs. 70.2% (*p* = 0.001; Figure 2A). Moreover, the overall mean of worse survival and 5-year SR were also observed for the patients with MACC1_high_ expression in either colon cancer (*n* = 328; mean OS: 84.0 vs. 92.1 months; *p* = 0.007; Figure 2B) or rectal cancers (*n* = 171; mean OS: 53.0 vs. 79.5 months; *p* = 0.019; Figure 2C).

In addition, we also analyzed the effects of the MACC1 index with cut-off on the CRC patient survival. Data revealed that worse survival had been observed for CRC patients with MACC1 above the cut-off than those with MACC1 below the cut-off (67.3 vs. 92.0 months; *p* < 0.001), and the 5-year SR for the two groups was 47.5 vs. 69.7% (*p* < 0.001; Supplementary Figure 1A). Similarly, worse survival and 5-year SR were also observed for the patients with MACC1 above the cut-off in either colon cancer (71.9 vs. 94.4 months; *p* < 0.001; Supplementary Figure 1B) or rectal cancers (43.8 vs. 75.0 months; *p* < 0.001; Supplementary Figure 1C).

**Figure 3:**
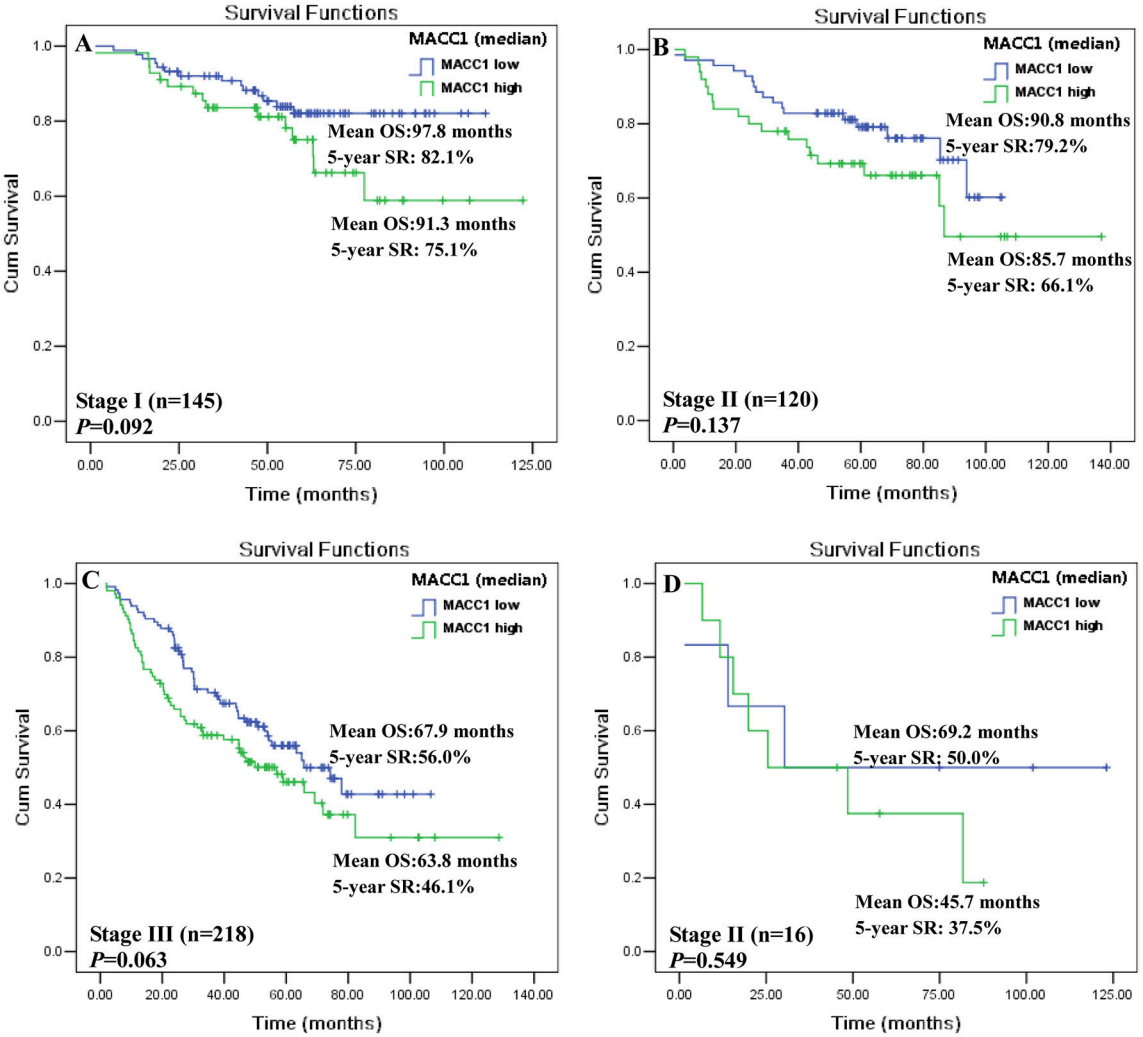
Kaplan-Meier survival analysis between MACC1_low_ and MACC1_high_ with the median (0.950) in distinct AJCC stage CRC patients. CRC patients with AJCC **(A)** stage I; **(B)** stage II; **(C)** stage III, and **(D)** stage IV.

To mitigate the biological and clinical heterogeneity of samples and patients, we further assessed the value of MACC1 index (median) for survival among patients with a particular AJCC stage I, II, III, and IV, respectively. Status of MACC1_high_ and MACC1_low_ can separate the Kaplan-Meier curves for patients with AJCC stage I (*n* = 145; 91.3 vs. 97.8 months; *p* = 0.092; Figure 3A), stage II (*n* = 120; 85.7 vs. 90.8 months; *p* = 0.137; Figure 3B), and stage III (*n* = 218; 63.8 vs. 67.9 months; *p* = 0.063; Figure 3C), respectively. Due to a limited size of the patients (*n* = 16), the survival analysis does not reach a statistic significance for patients with AJCC stage IV, though the survival of patients with MACC1_high_ was much shorter than those with MACC1_low_ (45.7 vs. 69.2 months; *p* = 0.549; Figure 3D). However, the threshold with cut-off value (MACC1_high_ *vs*. MACC1_low_) was dramatically associated with the patient survival in patients with AJCC stage I (*n* = 145; 68.0 vs. 105.0 months; *p* = 0.002; Supplementary Figure 2A), stage II (*n* = 120; 81.3 vs. 86.3 months; *p* = 0.035; Supplementary Figure 2B), and stage III (*n* = 218; 48.4 vs. 75.4 months; *p* = 0.002; Supplementary Figure 2C), respectively. Also, no statistical significance for patients with AJCC stage IV was observed (48.8 vs. 61.8 months; *p* = 0.773; Supplementary Figure 2D).

Next, we analyzed the prognostic value of MACC1 expression and other clinical parameters with the Cox's proportional hazards model. Data revealed that, in addition to subtypes of cancer and patient age, MACC1 index (media *n* = 0.950) is an independent prognostic factor for CRC patients (HR = 1.533, *p* = 0.005; Table 2). When the levels of MACC1 expression was grouped as above or below the cut-off levels, MACC1 expression status can also be an independent prognostic factor for CRC patient prognosis prediction (HR = 2.024, *p* < 0.001; Supplementary Table 1).

**Table 2:** Cox proportional hazards model analysis of variables affecting overall survival in colorectal cancer patients^*^.

**Variables**	**Categories**	**Univariate analysis**		**Multivariate analysis**	
		**HR (95% CI)**	***P***	**HR (95% CI)**	***P***
Cancer type	colon vs. rectal	1.237 (0.908–1.686)	0.178	1.311 (0.944–1.823)	0.106
Gender	male vs. female	0.908 (0.674–1.223)	0.525	0.907 (0.667–1.233)	0.534
Age (years)	>66 vs. ≤ 66	1.402 (1.045–1.880)	0.024	1.420 (1.054–1.910)	0.021
T category	T_3+4_ vs. T_1+2_	1.706 (1.252–2.323)	0.001	1.600 (1.155–2.215)	0.005
N category	N_1+2_ vs. N_0_	2.373 (1.757–3.207)	< 0.001	0.317 (0.088–1.137)	0.078
M category	M_1_ vs. M_0_	2.206 (1.069–3.838)	0.030	0.845 (0.307–2.326)	0.744
AJCC stage	III/IV vs. I/II	2.652 (1.952–3.604)	< 0.001	7.225 (1.930–27.04)	0.003
MACC1(median)^**^	high vs. low	1.165 (1.231–2.221)	0.001	1.533 (1.137–2.065)	0.005

*HR, hazard ratio; 95% CI, 95% confidence interval*.

* *Using Cox proportional hazard analysis, multivariate models were covariate adjusted for cancer type, gender, age, TNM, AJCC stage and MACC1 status*.

** *Median = 0.950 for MACC1 high or low*.

### Prognostic Stratification Effects of MACC1 Levels in CRC Patients

Moreover, we analyzed the prognostic stratification effects of MACC1 index (median) on various clinical parameters including sub-histological tumor type, gender of the patient and age, TNM status and AJCC stages. The data demonstrated that MACC1_high_ and MACC1_low_ is of great power in survival when these variables were stratified. As shown in Table 3, MACC1_high_ and MACC1_low_ can further notably separate the survival curve among patients with colon (*p* < 0.001) or rectal cancer (*p* = 0.017), male (*p* = 0.006) or female (*p* = 0.033), elder (*p* = 0.009) or younger (*p* = 0.046), T_1+2_ (*p* = 0.038) or T_3+4_ (*p* = 0.014), N_0_ (*p* = 0.012) or N_1+2_ (*p* = 0.048), M_0_ (*p* = 0.002), AJCC_I+II_ (*p* = 0.022), or AJCC_III+IV_ (*p* = 0.041). To be noted, the stratification survival analysis does not reach a statistical significance for patients with status of M_1_ (*p* = 0.549), which may be due to the limited size of only 16 patients. However, an indisputable trend was observed for the MACC1_high_ toward a worse outcome to stratified clinical parameters. Similar findings were obtained with the threshold value of MACC1 index with the cut-off (Supplementary Table 2).

**Table 3:** Log-rank Mantel-Cox analysis of stratified variables in survival by tumor MACC1 status (median = 0.95) in CRC patients^*^.

**Variables**	**Stratified variables**	**Whole cohort**				**MACC1**_****low****_			**MACC**_****high****_			
		**No. total**	**No. events**	**Survival mean (95% CI)**	***p***	**No. total**	**No. events**	**Survival mean (95% CI)**	**No. total**	**No. events**	**Survival mean (95% CI)**	***p***
All CRC	/	499	180	90.7 (85.2–96.2)		280	85	90.4 (84.5–96.3)	219	95	80.1 (71.6–88.7)	0.001
Cancer types	Colon	328	117	92.8 (86.4–99.3)	0.177	173	52	92.1 (85.1–99.2)	155	65	84.1 (74.3–93.9)	0.007
	Rectal	171	63	70.4 (62.8–78.0)		107	33	79.5 (71.9–87.1)	64	30	54.2 (46.6–61.7)	0.019
Gender	Male	286	107	82.4 (76.3–88.4)	0.525	171	54	78.9 (73.2–84.6)	115	53	71.8 (62.0–81.6)	0.006
	Female	213	73	91.8 (83.4–100)		109	31	91.7 (82.4–101)	104	42	82.9 (70.3–95.5)	0.033
Age	≤ 66 years	260	84	95.4 (88.0–103)	0.023	153	41	95.3 (87.9–103)	107	43	82.4 (70.3–94.5)	0.009
	>66 years	239	96	81.8 (74.4–89.2)		127	44	76.1 (65.7–86.4)	112	52	74.8 (67.8–81.9)	0.046
Tumor status	T_1+2_	221	61	92.9 (86.5–99.3)	0.001	132	30	99.7 (92.3–107)	89	31	83.2 (72.6–93.9)	0.038
	T_3+4_	278	119	82.1 (74.6–89.7)		148	55	74.1 (67.6–80.5)	130	64	72.9 (61.7–84.1)	0.014
Nodal status	N_0_	271	70	103.0 (96.0–110)	< 0.001	161	33	92.8 (87.0–98.6)	110	37	92.2 (80.2–104)	0.012
	N_1+2_	228	110	72.0 (64.5–79.6)		119	52	76.1 (66.7–85.6)	109	58	64.6 (53.5–75.7)	0.048
Metastasis status	M_0_	483	170	91.6 (86.0–97.2)	0.027	274	82	84.0 (78.9–89.0)	209	88	81.7 (73.0–89.0)	0.002
	M_1_	16	10	59.6 (35.1–84.1)		6	3	69.2 (25.5–112)	10	7	45.7 (25.4–66.1)	0.549
AJCC stage	I+II	265	64	105.3 (98.2–112)	< 0.001	159	31	95.7 (84.0–99.4)	106	33	93.7 (88.0–99.4)	0.022
	III+IV	234	116	70.2 (62.7–77.6)		121	54	75.2 (65.9–84.6)	113	62	62.2 (51.4–72.9)	0.041

*95% CI, 95% confidence interval; TNM, lymph-node-metastasis and stage according to the 7th TNM staging system*.

* *Among the whole cohort of the 503 CRC patients, follow-up data were available for 499 patients till the last follow-up*.

## Discussion

MACC1 gene was first identified by Stein et al. (4) in 2009 in patients with colon cancer. Thereafter, a wealth of studies have been carried out in variety of malignancies, strengthening the potential application of both MACC1 transcripts and protein expression as a novel prognostic indicator, and MACC1 as a therapeutic target was recommended for cancers (21). The relevance of MACC1 expression including its genetic and proteomic has been explored in a large body of studies. In this context, higher levels of circulating MACC1 mRNA, peripheral soluble or tumor lesion protein expression was significantly associated with poor survival in patients such as lung cancer (22, 23), gastric cancer (24), glioma (25), cervical cancer (26), hepatocellular, and renal pelvis carcinoma (12, 27).

Mechanisms of MACC1 in cancer development and progression have been reported in *in vitro* cell model and pre-clinical murine models. MACC1 has been found to enhance gastric tumor cell migration, invasion and epithelial-mesenchymal transition (EMT) *in vitro* (28). Authors further addressed that MACC1 overexpression favors tumor growth and promotes tumor metastasis in an athymic mice model. Multiple signal pathways such as various microRNA (29, 30), lncRNA (31, 32), circular RNA (33), and molecules such as deleted in breast cancer 1 (34), statin and rottlerin (35), have been observed in the regulation of MACC1 expression. Based on these findings, MACC1 as a potential valuable therapeutic target has been proposed.

In the current study, we evaluated the prognostic significance of tumor lesion MACC1 expression in 503 CRC patients. Our data revealed that MACC1_high_ is strongly associated with poor disease outcome and can be an independent prognostic factor. More importantly, MACC1 status could further improve the prognostic power for stratified clinical parameters, such as basic patient characteristics (patient age and gender) or distinct pathological factors (cancer subtype, TNM status and AJCC stages). Our data clearly demonstrated that, patients with MACC1_high_ have a significantly shorter survival rate than those with MACC1_low_ in various stratified parameters. These parameters include patient age, gender, colon or rectal cancer, TNM status and AJCC stage. To be noted, our results showed that threshold with the cut-off value based on ROC was much powerful in association with CRC patient survival than that of the median of the MACC1 index. As Rohr et al. (20) suggested in their recent study that the cut-off determination by ROC is an important step to ensure the future use of MACC1 protein expression, and that this can be more easily adapted to clinical practice. However, this may cause bias due to the ROC analysis and the prognostic power of the MACC1 expression in CRC patients were performed on the same cohort.

Moreover, our data revealed that the percentage of MACC1_high_ was more frequently observed in colon cancer patients. Patients with MACC1_high_ expression in both colon cancer and rectal cancer showed a significantly worse prognosis than those with MACC1_low_. In line with this, similar findings have been obtained by Zhu et al. (36) indicate that the overexpression of MACC1 was associated with poor survival in patients with colonic adenocarcinoma, and that MACC1 status can be an independent predictor of prognosis in patients with colonic adenocarcinoma. Moreover, a study by Rohr et al. (20) recently presented that status of MACC1 expression could stratify stage II colon cancer patients with unfavorable proficient mismatch repair (pMMR) status, and a distinct stage II colon cancer patients with pMMR/MACC1_low_ had a favorable prognosis similar to those with deficient mismatch repair (dMMR). In breast cancer, when estrogen receptor (ER) status were stratified, MACC1 was of prognostic value for both ER-negative and ER-positive patients (37). However, more investigation is needed to solidify the significance of the prognostic stratification of MACC1 expression in cancers.

In conclusion, we provided the evidence that tumor lesion MACC1 status is a clinical prognostic biomarker for patients with CRC and that it is also an improved prognostic significance for distinct stratified clinical parameters.

## Author Contributions

AL and W-HY study design. XZ and AL performed experiments. R-LZ, J-GZ, and X-FH material support and data acquisition. AL and W-HY performed statistical analysis and drafted the manuscript. All authors read and approved the final manuscript.

### Conflict of Interest Statement

The authors declare that the research was conducted in the absence of any commercial or financial relationships that could be construed as a potential conflict of interest.

## Supplementary Material

Supplementary Figure 1 Kaplan-Meier survival analysis of the index of MACC1 expression with the cut-off (1.04) for CRC patients. Comparison of overall survival between MACC1 below the cut-off and MACC1 above the cut-off among **(A)** all CRC patients; **(B)** colon cancer patients; and **(C)** rectal cancer patients.

Supplementary Figure 2 Kaplan-Meier survival analysis between MACC1_low_ and MACC1_high_ with the cut-off (1.04) in distinct AJCC stage CRC patients. CRC patients with AJCC **(A)** stage I; **(B)** stage II; **(C)** stage III, and **(D)** stage IV.

Supplementary Table 1 Multivariate analysis of prognostic factors for overall survival in colorectal cancer patients.

Supplementary Table 2 Log-rank Mantel-Cox analysis of stratified variables in survival by tumor MACC1 status (Cut-off = 1.04) in CRC patients.
