# Supplementary figures and images for: Prognostic and Risk Stratification Value of Lesion MACC1 Expression in Colorectal Cancer Patients
Source: Front Oncol. 2019 Feb 5;9:28. doi: 10.3389/fonc.2019.00028 (PMC6371040; doi:10.3389/fonc.2019.00028)

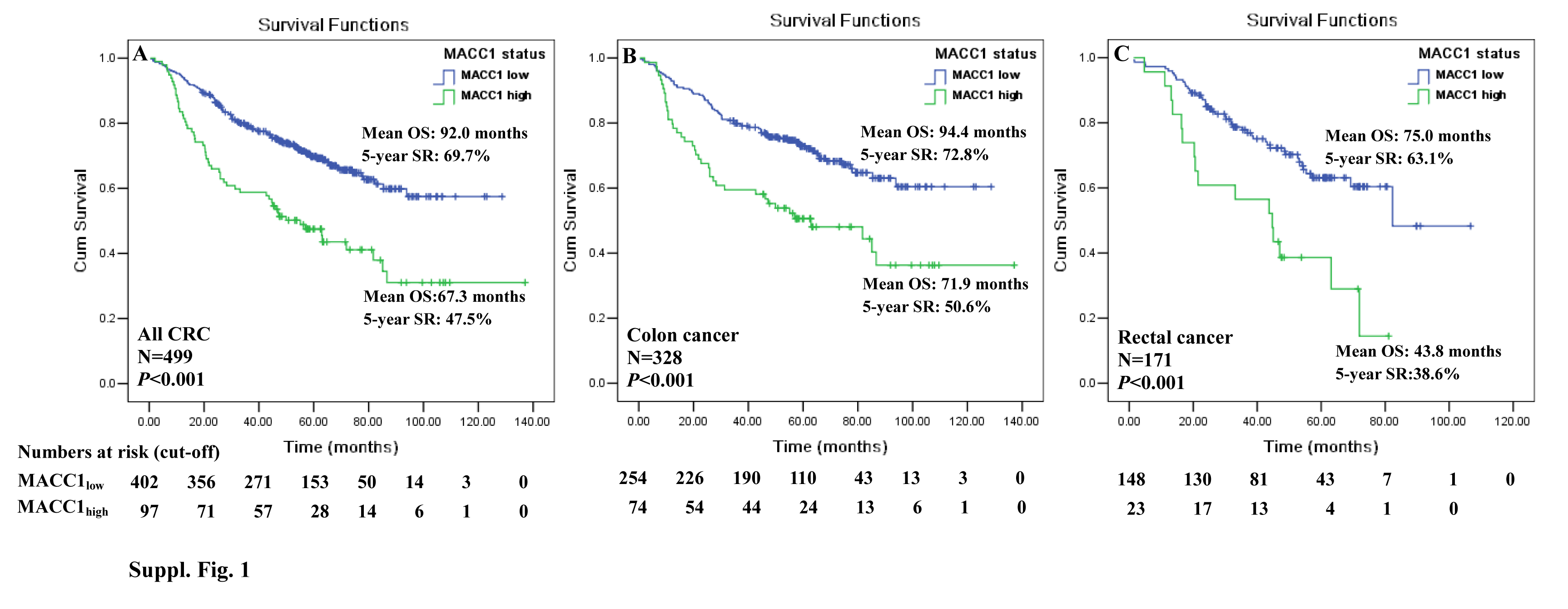

Supplement: Supplementary Figure 1 — Kaplan-Meier survival analysis of the index of MACC1 expression with the cut-off (1.04) for CRC patients. Comparison of overall survival between MACC1 below the cut-off and MACC1 above the cut-off among (A) all CRC patients; (B) colon cancer patients; and (C) rectal cancer patients. [file Image_1.TIF]

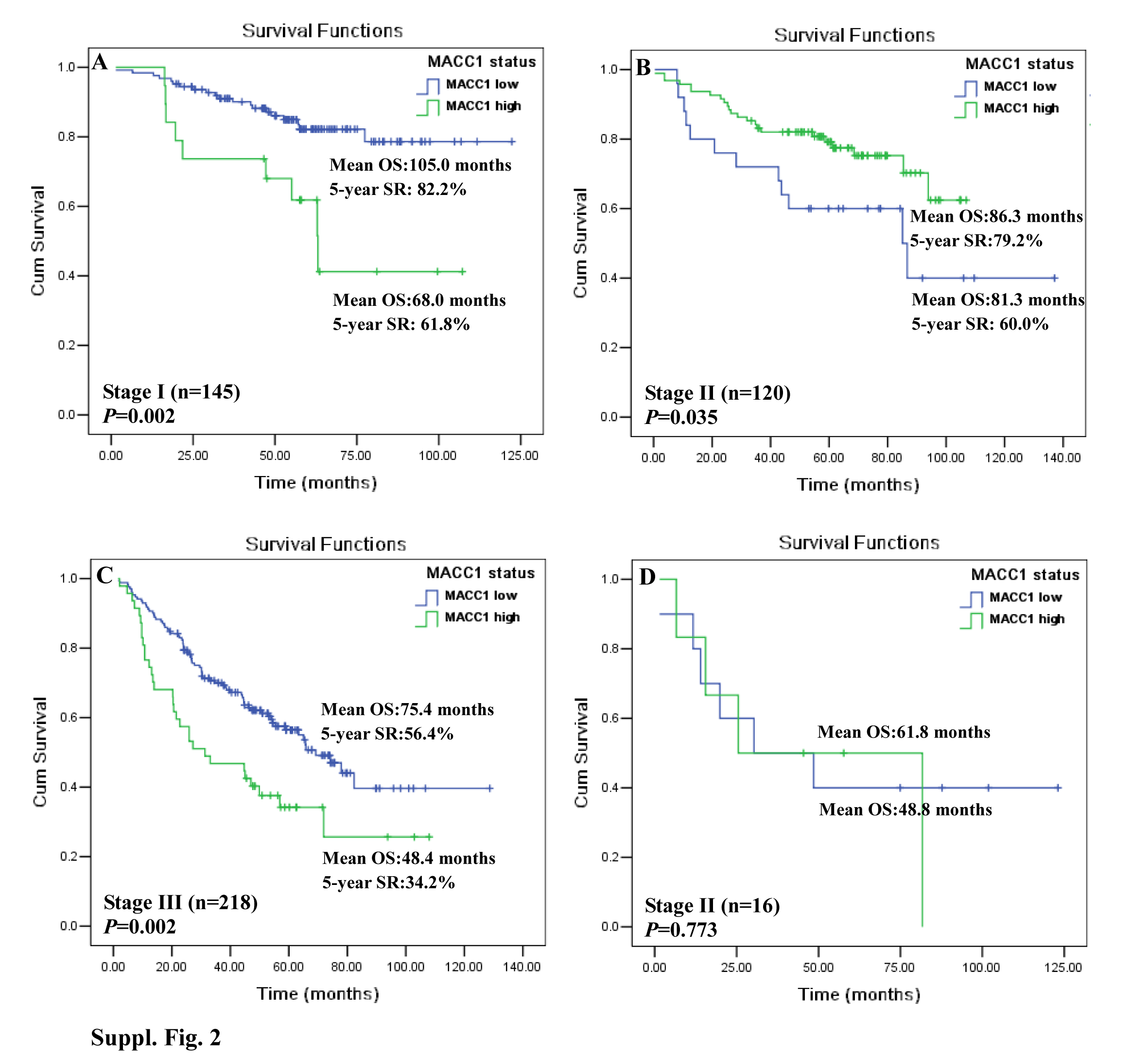

Supplement: Supplementary Figure 2 — Kaplan-Meier survival analysis between MACC1low and MACC1high with the cut-off (1.04) in distinct AJCC stage CRC patients. CRC patients with AJCC (A) stage I; (B) stage II; (C) stage III, and (D) stage IV. [file Image_2.TIF]
