# Supplementary material for: Prognostic and Risk Stratification Value of Lesion MACC1 Expression in Colorectal Cancer Patients
Source: Front Oncol. 2019 Feb 5;9:28. doi: 10.3389/fonc.2019.00028 (PMC6371040; doi:10.3389/fonc.2019.00028)
Supplement: Supplementary Table 1 — Multivariate analysis of prognostic factors for overall survival in colorectal cancer patients. [file Table_1.DOC]

| **Suppl. Table 1**  Multivariate analysis of prognostic factors for overall survival in colorectal cancer patients**^1^** | | | | | | | |
| --- | --- | --- | --- | --- | --- | --- | --- |
| Variables | Categories |  | Univariate Analysis | |  | Multivariate Analysis | |
|  |  |  | HR (95% CI) | *P* |  | HR (95% CI) | *P* |
| Cancer type | colon *vs* rectal |  | 1.237 (0.908 – 1.686) | 0.178 |  | 1.398 (1.004-1.947) | 0.048 |
| Gender | male *vs* female |  | 0.908 (0.674 – 1.223) | 0.525 |  | 0.960 (0.706-1.305) | 0.795 |
| Age (years) | >66 *vs* ≤66 |  | 1.402 (1.045 -1.880) | 0.024 |  | 1.415 (1.049 – 1.910) | 0.023 |
| T category | T_3+4_ *vs* T_1+2_ |  | 1.706 (1.252 – 2.323) | 0.001 |  | 1.354 (0.916 – 2.003) | 0.129 |
| N category | N_1+2_ *vs* N_0_ |  | 2.373 (1.757 – 3.207) | <0.001 |  | 1.350 (0.629 – 2.897) | 0.441 |
| M category | M_1_ *vs* M_0_ |  | 2.206 (1.069 – 3.838) | 0.030 |  | 1.105 (0.408 – 2.992) | 0.844 |
| AJCC stage | III/IV *vs* I/II |  | 2.652 (1.952 – 3.604) | <0.001 |  | 1.394 (0.809 – 2.401) | 0.231 |
| MACC1**^*^** | high *vs* low |  | 2.200 (1.604 – 3.017) | <0.001 |  | 2.024 (1.468 – 2.790) | <0.001 |
| Abbreviations: HR=hazard ratio; 95% CI=95% confidence interval. **^*^** Cut-off=1.04 for MACC1 high or low.  **^1^**Using Cox proportional hazard analysis, multivariate models were covariate adjusted for cancer type, gender, age, TNM, AJCC stage and MACC1 status. | | | | | | | |
