# Supplementary material for: Prognostic and Risk Stratification Value of Lesion MACC1 Expression in Colorectal Cancer Patients
Source: Front Oncol. 2019 Feb 5;9:28. doi: 10.3389/fonc.2019.00028 (PMC6371040; doi:10.3389/fonc.2019.00028)
Supplement: Supplementary Table 2 — Log-rank Mantel-Cox analysis of stratified variables in survival by tumor MACC1 status (Cut-off = 1.04) in CRC patients. [file Table_2.DOC]

| **Suppl. Table 2** Log-rank Mantel-Cox analysis of stratified variables in survival by tumor MACC1 status **(**Cut-off=1.04) in CRC patients***** | | | | | | | | | | | | | | | | |
| --- | --- | --- | --- | --- | --- | --- | --- | --- | --- | --- | --- | --- | --- | --- | --- | --- |
| Variables | Stratified variables | Whole cohort | | | |  | MACC1**_low_** | | | |  | MACC**_high_** | | | |  |
|  |  | No.  Total | No. Events | Survival  Mean (95% CI) | *p* |  | No.  Total | No. Events | Survival  Mean (95% CI) | |  | No.  Total | No. Events | Survival  Mean (95% CI) | | *p* |
| All CRC | / | 499 | 180 | 90.7 (85.2-96.2) |  |  | 402 | 124 | 92.0(86.7-97.5) | |  | 97 | 56 | 68.0(56.4-79.7) | | <0.001 |
|  |  |  |  |  |  |  |  |  |  | |  |  |  |  | |  |
| Cancer types | colon | 328 | 117 | 92.8 (86.4-99.3) | 0.177 |  | 254 | 77 | 94.4 (88.0-101) | |  | 74 | 40 | 71.9 (58.4-85.5) | | <0.001 |
|  | rectal | 171 | 63 | 70.4 (62.8-78.0) |  |  | 148 | 47 | 75.0(66.8-83.2) | |  | 23 | 16 | 43.8 (32.8-54.7) | | <0.001 |
|  |  |  |  |  |  |  |  |  |  | |  |  |  |  | |  |
| Gender | Male | 286 | 107 | 82.4 (76.3-88.4) | 0.525 |  | 234 | 78 | 86.5(80.0-93.1) | |  | 52 | 29 | 60.5 (48.5-72.5) | | 0.002 |
|  | Female | 213 | 73 | 91.8 (83.4-100) |  |  | 168 | 46 | 95.1(86.7-104) | |  | 45 | 27 | 64.0 (47.5-80.5) | | <0.001 |
|  |  |  |  |  |  |  |  |  |  |  |  |  |  |  |  |  |
| Age | ≤66ys | 260 | 84 | 95.4 (88.0-103) | 0.023 |  | 213 | 55 | 94.8(88.3-101) | |  | 47 | 29 | 62.3 (46.4-78.2) | | <0.001 |
|  | ＞66ys | 239 | 96 | 81.8 (74.4-89.2) |  |  | 189 | 69 | 84.5(75.7-93.2) | |  | 50 | 27 | 62.8 (50.7-75.0) | | 0.044 |
|  |  |  |  |  |  |  |  |  |  |  |  |  |  |  |  |  |
| Tumor status | T_1+2_ | 221 | 61 | 92.9 (86.5-99.3) | 0.001 |  | 190 | 45 | 97.1(90.4-104) | |  | 31 | 16 | 64.1(49.7-78.4) | | 0.002 |
|  | T_3+4_ | 278 | 119 | 82.1 (74.6-89.7) |  |  | 121 | 79 | 84.7(76.9-92.5) | |  | 66 | 40 | 63.1(49.0-77.2) | | 0.001 |
|  |  |  |  |  |  |  |  |  |  |  |  |  |  |  |  |  |
| Nodal status | N_0_ | 271 | 70 | 103.0 (96.0-110) | <0.001 |  | 224 | 46 | 99.6(93.6-106) | |  | 47 | 24 | 77.2(61.0-93.4) | | <0.001 |
|  | N_1+2_ | 228 | 110 | 72.0 (64.5-79.6) |  |  | 178 | 78 | 76.5(67.9-85.1) | |  | 50 | 32 | 49.9(37.6-62.1) | | 0.004 |
|  |  |  |  |  |  |  |  |  |  |  |  |  |  |  |  |  |
| Metastasis  status | M_0_ | 483 | 170 | 91.6 (86.0-97.2) | 0.027 |  | 392 | 118 | 92.5(87.0-98.2) | |  | 91 | 52 | 68.7(55.6-80.8) | | <0.001 |
|  | M_1_ | 16 | 10 | 59.6 (35.1-84.1) |  |  | 10 | 6 | 61.8(30.0-93.7) | |  | 6 | 4 | 48.8(18.0-79.6) | | 0.773 |
|  |  |  |  |  |  |  |  |  |  |  |  |  |  |  |  |  |
| AJCC stage | I+II | 265 | 64 | 105.3 (98.2-112) | <0.001 |  | 221 | 43 | 101(94.6-107) | |  | 44 | 21 | 81.5(64.6-98.4) | | <0.001 |
|  | III+IV | 234 | 116 | 70.2 (62.7-77.6) |  |  | 181 | 81 | 75.6(67.1-84.1) | |  | 53 | 35 | 48.2(36.8-59.5) | | 0.002 |
| Abbreviations: 95% CI=95% confidence interval; TNM, lymph-node-metastasis and stage according to the 7^th^ TNM staging system.  *****Among the whole cohort of the 503 CRC patients, follow-up data were available for 499 patients till the last follow-up. | | | | | | | | | | | | | | | | |
